# A case of hereditary alpha tryptasemia and presumptive eosinophilic granulomatosis with polyangiitis

**DOI:** 10.1016/j.jacig.2025.100481

**Published:** 2025-04-22

**Authors:** Sonia Iqbal, Joseph A. Baxter, Karla E. Adams

**Affiliations:** a Department of Internal Medicine, Malcolm Grow Medical Center, Joint Base Andrews, Md; b Department of Allergy and Immunology, 56th Medical Group, Luke Air Force Base, Ariz; c Department of Allergy and Immunology, Wilford Hall Ambulatory Surgical Center, Lackland Air Force Base, Tex

**Keywords:** Hereditary alpha tryptasemia, eosinophilic granulomatosis with polyangiitis, hypereosinophilia, mepolizumab, elevated tryptase level

## Abstract

This case is notable for the potential overlap of hereditary alpha tryptasemia and eosinophilic granulomatosis with polyangiitis. The successful use of mepolizumab, initially for eosinophilic granulomatosis with polyangiitis but potentially benefiting the patient's hereditary alpha tryptasemia, offers a novel approach to managing complex cases involving both rare disorders.

A 50-year-old male with a history of gastroesophageal reflux disease presented with a 3-month history of chronic cough along with intermittent dyspnea and wheezing, which became worse at night, as well as urticaria and pruritus, with no improvement after taking an intranasal corticosteroid, proton pump inhibitor, and oral antihistamine. He did not endorse significant neurologic symptoms. After acute worsening of his cough and dyspnea 1 month after several outpatient clinic and emergency room encounters, he was hospitalized for coronavirus disease 2019 (COVID-19) pneumonia and treated with remdesivir and dexamethasone. However, because of persistent symptoms, he presented to the pulmonary department for further evaluation. His laboratory testing results included elevated absolute eosinophil counts (1000-1700 cells/μL) that persisted for 6 months after hospitalization.

Hypereosinophilia (eosinophil count >1500 cells/μL) provoked a multidisciplinary workup involving the allergy/immunology, pulmonary, infectious disease, and hematology/oncology departments. Given his recent travel to Guam at symptom onset, he underwent an infectious workup, the results of which were negative for Strongyloides IgG, as well as 3 stool studies for ova and parasites. A bone marrow biopsy was negative for primary hematologic process, with negative *JAK2*, c-KIT, BCR/ABL, FIP1L1/CHIC2/PDGFRA FISH probe results, T-cell gene rearrangement study results, and no atypical mast cells in his bone marrow. High-resolution chest computed tomography (Fig 1) showed ground glass opacities in the right upper central lobe with associated reticulation and traction bronchiectasis. Bronchoscopy with lavage was performed; the results were unremarkable for any bacterial, fungal, or malignant cells, with approximate cell differential results as follows (all of 100%): macrophage count, 70%; lymphocyte count, 18%; eosinophils 7%; and neutrophil count, 5%. The results of additional laboratory workup (including antineutrophil cytoplasmic antibody testing, a fungal IgE panel, and an aeroallergen panel) were also negative; his total IgE level was 551 IU/mL, and his vitamin B_12_ level was 570 pg/mL (232-1245 pg/mL). Testing for alpha-1 antitrypsin deficiency mutation yielded a normal result. However, his baseline serum tryptase levels were elevated at 23 and 17 ng/mL (2.2-13.2 ng/mL). The results of tryptase copy number variation testing (*TPSAB1* copy number variation) were significant for an extra allelic alpha-tryptase at *TPSAB1,* with a calculated alpha-tryptase copy number of 3 confirming diagnosis of hereditary alpha tryptasemia (HαT).

**Fig 1:**
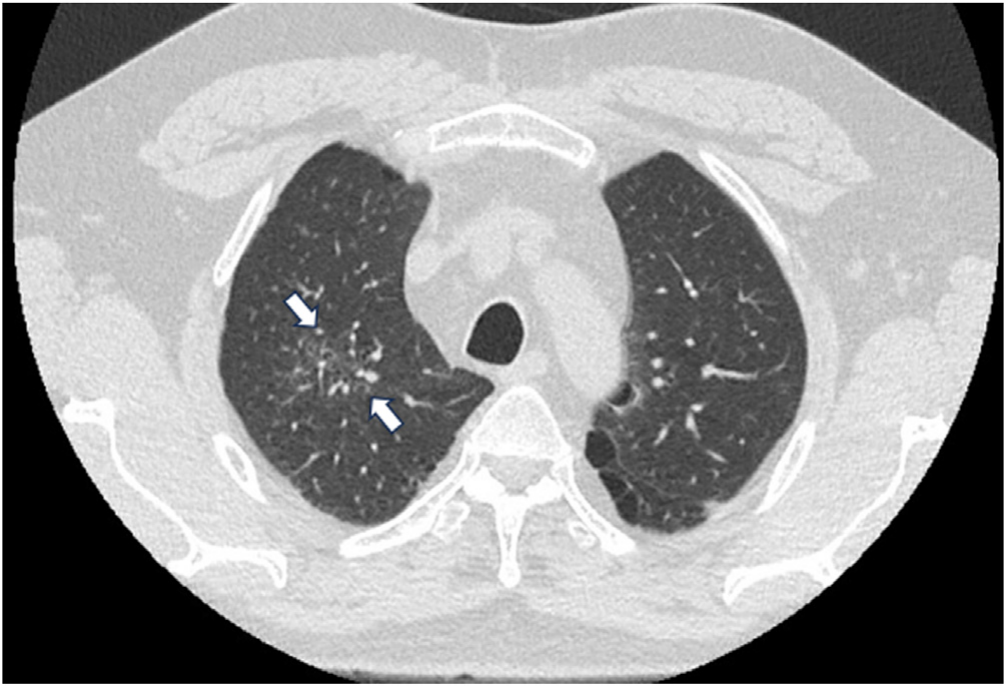
Chest computed tomography scan without contrast. Central right upper lobe ground glass opacity with associated reticulation and traction bronchiectasis (*indicated by white arrows*).

Although the results of spirometry were negative (FEV_1_ value, 3.58 L [reference = 3.77 L]; ratio of FEV_1_ value to forced vital capacity of 76% measured and 80% predicted; no significant bronchodilator response; and normal diffusion capacity of lung for carbon monoxide), a positive methacholine challenge result (25% reduction in FEV_1_ value after incremental doses of methacholine of ≤8 mg/mL) aided in diagnosis of severe persistent asthma. The patient was on step 6 of treatment (detailed in the remainder of this paragraph) per the National Asthma Education and Prevention Program 2020 guidelines with a high-dose inhaled corticosteroid/long-acting β-agonist and oral corticosteroids. A transthoracic echocardiogram showed normal left ventricular systolic function with no significant valvular disease. The patient had no cardiac, renal, or gastrointestinal involvement to raise suspicion for other autoimmune disease. Furthermore, a presumptive diagnosis of eosinophilic granulomatosis with polyangiitis (EGPA) was made on the basis of the criteria of obstructive airway disease and a blood eosinophil count higher than 1 × 10^3^/μL per the American College of Rheumatology classification. EGPA is a small-to-medium vessel vasculitis with criteria for diagnosis that may include obstructive airway disease, nasal polyps, mononeuritis multiplex, a blood eosinophil count higher than 1 × 10^3^/μL, and extravascular eosinophilic-predominant inflammation on biopsy.^1^ Eosinophilic asthma is another differential diagnosis to consider, given peripheral eosinophilia and a sputum eosinophil level greater than 3%.^2^ Although diagnosis of EGPA without biopsy is difficult, biopsy was deferred given the patient’s overall clinical improvement and because it would not change management. The patient began receiving prednisone, 40 mg daily, and mepolizumab, 300 mg monthly (with gradual taper off oral corticosteroids once his clinical condition improved), as well as a daily regimen of fluticasone/umeclidinium/vilanterol (he was previously taking fluticasone/salmeterol and tiotropium, but this was consolidated to triple therapy to aid with medication compliance), cetirizine, montelukast, and albuterol as needed.

HαT is an autosomal dominant genetic trait defined by 1 or more extra copies of the α-tryptase allele in the *TPSAB1* locus; it is found in approximately 1 in 20 White individuals.^3^ Most affected individuals have baseline tryptase levels higher than 8 ng/mL.^4^ HαT is assessed by only 1 laboratory through droplet digital PCR assay. Clinical presentation may include recurrent cutaneous pruritus and flushing, gastric reflux, irritable bowel syndrome, chronic arthralgias and myalgias, joint hypermobility, dysautonomia, congenital skeletal abnormalities, retained primary teeth, and Hymenoptera allergy.^5^ Antihistamine therapy targeting both H1 and H2 receptors for cutaneous and gastrointestinal symptoms has been recommended, but there are no prospective studies to support more targeted treatment.^5^

Mepolizumab (a mAB that inhibits IL-5 signaling), which is the only US Food and Drug Administration–approved mAb treatment for EGPA, was shown in a recent randomized, placebo-controlled, double-blind clinical trial to achieve remission in patients with relapsing or refractory EGPA.^6^ The plan with mepolizumab was to wean the patient from EGPA dosing (300 mg) to asthma dosing (100 mg) every 4 weeks and consider the disease in remission if the patient’s symptoms remained controlled. Small studies have shown the step-down approach to be effective.^7^ mAbs are approved for the treatment of moderate-to-severe asthma, and more investigation is needed to show benefit for management of HαT. Another case involved a pediatric patient with HαT in whom successful treatment was achieved with omalizumab.^8^ The patient’s earlier presentation of urticaria, pruritus, and chronic gastric reflux may have been attributed to HαT. However, there was no reported improvement of these symptoms after he began taking mepolizumab. Although whether his symptomatology was due to both conditions or whether the finding of HαT was coincidental is unclear, ultimately, a multimodal approach personalized to the individual patient likely resulted in the best outcomes.

This case contributes to the literature by describing a rare case of suspected EGPA and HαT. Although uncommon, they can contribute to significant morbidity as outlined in this case report. Further research is needed to clarify the clinical significance of these 2 disease processes when concurrently present and develop guidelines regarding early diagnosis and effective management.

Informed consent statement: Informed consent was obtained from the patient for the publication of this case report, including any accompanying images and clinical information.

## Disclosure statement

Disclosure of potential conflict of interest: The authors declare that they have no relevant conflicts of interest.
